# Optimizing prediction of binge eating episodes: a comparison approach to test alternative conceptualizations of the affect regulation model

**DOI:** 10.1186/s40337-014-0028-9

**Published:** 2014-09-14

**Authors:** Matthew Fuller-Tyszkiewicz, Ben Richardson, Helen Skouteris, David Austin, David Castle, Lucy Busija, Britt Klein, Millicent Holmes, Jaclyn Broadbent

**Affiliations:** School of Psychology, Deakin University, 221 Burwood Highway, Burwood, VIC 3125 Australia; Psychiatry Department, University of Melbourne, Melbourne, VIC Australia; St Vincent’s Hospital, Melbourne, VIC Australia; Faculty of Health, Deakin University, Burwood, Australia; DVC-Research & Innovation Portfolio; the School of Health Sciences; and the Collaborative Research Network, Federation University, Ballarat, Australia; National Institute for Mental Health Research, The Australian National University, Canberra, Australia

**Keywords:** Threshold modeling, Binge eating, Negative mood, Intensive longitudinal design, Experience sampling

## Abstract

**Background:**

Although a wealth of studies have tested the link between negative mood states and likelihood of a subsequent binge eating episode, the assumption that this relationship follows a typical linear dose–response pattern (i.e., that risk of a binge episode increases in proportion to level of negative mood) has not been challenged. The present study demonstrates the applicability of an alternative, non-linear conceptualization of this relationship, in which the strength of association between negative mood and probability of a binge episode increases above a threshold value for the mood variable relative to the slope below this threshold value (threshold dose response model).

**Methods:**

A sample of 93 women aged 18 to 40 completed an online survey at random intervals seven times per day for a period of one week. Participants self-reported their current mood state and whether they had recently engaged in an eating episode symptomatic of a binge.

**Results:**

As hypothesized, the threshold approach was a better predictor than the linear dose–response modeling of likelihood of a binge episode. The superiority of the threshold approach was found even at low levels of negative mood (3 out of 10, with higher scores reflecting more negative mood). Additionally, severity of negative mood beyond this threshold value appears to be useful for predicting time to onset of a binge episode.

**Conclusions:**

Present findings suggest that simple dose–response formulations for the association between negative mood and onset of binge episodes miss vital aspects of this relationship. Most notably, the impact of mood on binge eating appears to depend on whether a threshold value of negative mood has been breached, and elevation in mood beyond this point may be useful for clinicians and researchers to identify time to onset.

## Background

Binge eating, which is characterized by rapid overconsumption of food and a sense of loss of control over eating [1], is a common feature of eating disorders, such as bulimia nervosa, anorexia nervosa-binge/purge subtype, and eating disorder not otherwise specified. Furthermore, it is the key symptom of Binge Eating Disorder, recently defined in the DSM-V [2]. Given its known links to a range of health consequences, including obesity, gastrointestinal problems, and Type II diabetes [3,4], understanding of the antecedents (both distal and proximate) for binge eating is crucial for developing effective treatment solutions. This study proposes a novel modeling technique (threshold modeling) that may be used to better understand the role that negative mood states play in the onset of binge eating episodes.

**Figure 1:**
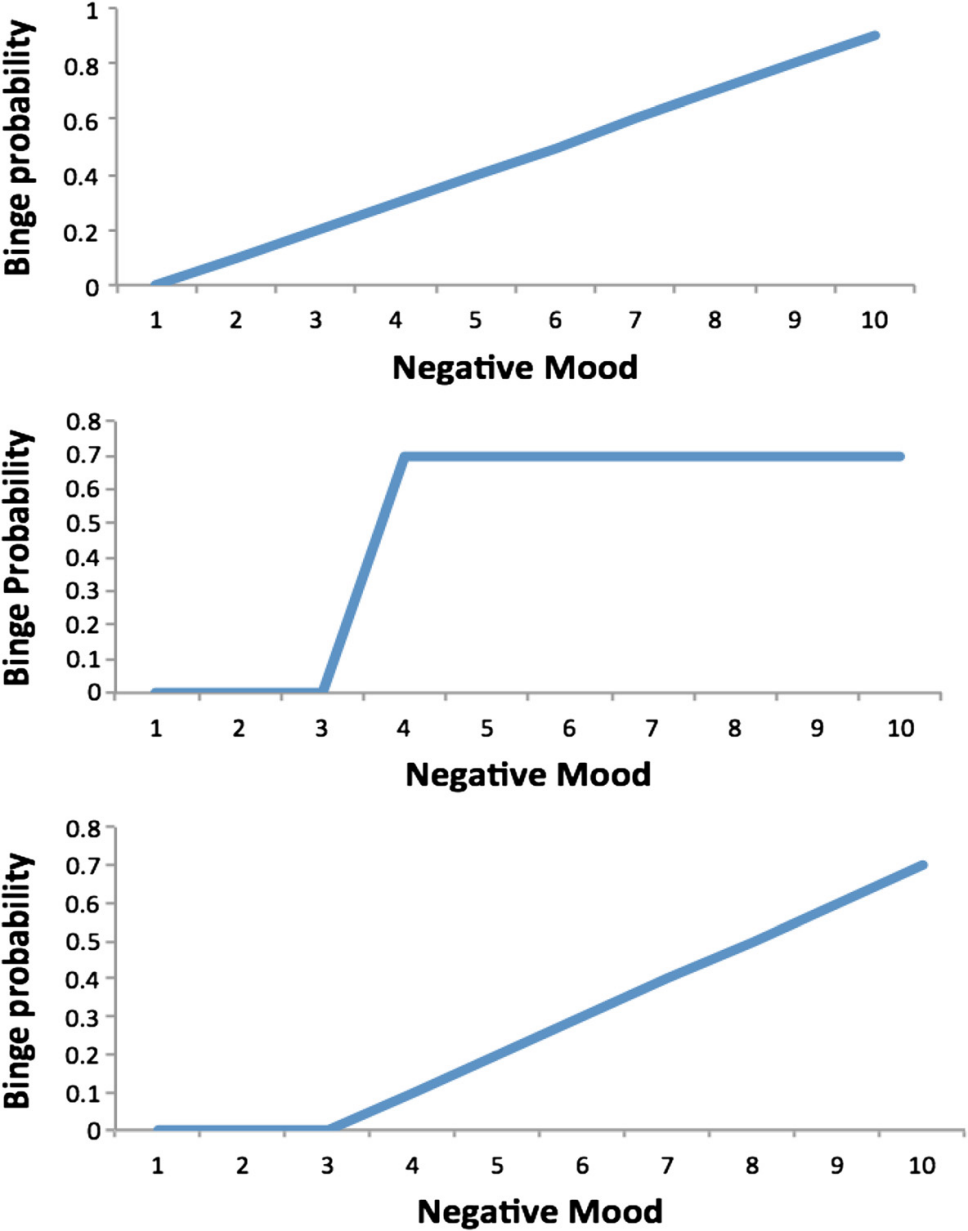
**Alternative conceptualisations of the relationship between negative mood and likelihood of a binge eating episode.**

The affect regulation model [5,6] proposes that individuals binge eat in order to reduce severe and potentially chronic states of negative mood (e.g., through distraction or release of neurotransmitters, such as serotonin, that alleviate negative mood). One way of interpreting this model is as a linearized relationship, such that as the severity of negative mood increases, so too will the likelihood of a binge episode (see the top panel of Figure 1). Indeed, this is the most common modeling approach in the literature, and these studies have generally found negative mood severity to be predictive of a binge event [5].

However, the relationship between negative mood and risk of binge eating predicted in the affect regulation model does not necessarily have to be linear. In particular, it could be that there is some level or ‘threshold’ of negative affect that must be surpassed before a coping response (i.e., binge eating) is triggered. That is, once a ‘comfortable’ level of negative mood is breached, the individual is highly likely to binge, whereas any negative mood state below this threshold is highly unlikely to produce a binge. Increases beyond this threshold may have one of several consequences. First, subsequent increases beyond the threshold may have negligible impact on likelihood of a binge as the act of crossing the threshold is decisive in predicting onset (we will call this the *threshold + stability model*; see middle panel in Figure 1). For instance, supposing that an individual’s threshold value is 3 out of 10 on a negative mood scale, a mood rating of 9 is just as likely (barring measurement error) to result in a binge as a mood rating of 4, since both exceed the level of negative mood with which the individual is comfortable. An alternative, and more probable, scenario is that the extent to which negative mood exceeds the threshold further increases the likelihood of a binge episode as there is greater impetus to reduce negative feelings (the *threshold + dose response model*; see bottom panel in Figure 1). To the authors’ knowledge, neither of these threshold approaches has been tested previously in relation to binge eating.

The nature of the mood-binge eating relationship is an important issue that has both theoretical and practical implications. For example, if either of the threshold models is correct, severity of negative mood may be more predictive of time to onset than simply likelihood of a binge episode. That is, although the relationship between negative mood and time to onset of a binge would be linear – negative mood steadily increases as time to onset reduces – the actual point at which the binge becomes likely occurs earlier, at a lower level of negative mood. This is important for timely intervention which could be cued to occur once the threshold of negative mood is surpassed rather than waiting until negative mood has increased to a high level, at which point the binge eating episode is imminent.

The present study used the experience sampling method (ESM [7]) to explore the nature of the negative mood-binge relationship by: (1) comparing the severity and threshold approaches for modeling the relationship between negative mood and binge eating, and (2) evaluating the extent to which severity of negative mood predicts time to onset of a binge episode. ESM was used in preference for experimental designs in order to monitor mood and binge eating symptoms in a more ecologically valid manner [8], and to also allow for multiple assessment points in order to better ascertain the time course from trigger (elevated negative mood) to event (binge episode) [8,9].

Superiority was operationalized as the model that exhibited the strongest effect of mood on binge eating. In addition, we predicted that shape of the severity-time to onset relationship would be negative and log-linear, such that more extreme negative mood would be associated with shorter times to onset for a binge.

## Methods

### Participants

Following ethics approval from the university’s Human Research Ethics Committee, participants were recruited via brief presentations at the beginning of tutorials and lectures in numerous classes at a large metropolitan university in Melbourne, Australia. The presentations emphasized that the study aim was to evaluate mood and eating experiences of women over a typical week. Participation was not limited to those with a current diagnosis or prior history of an eating disorder. Recruitment was limited to women in order to be consistent with the majority of prior studies investigating the affect regulation model from an experience sampling framework [5].

A total of 93 women agreed to participate, which is considerably greater than the average number of participants in previous ESM studies (Mean number = 28.7 participants, [5]). They had an age range of 18 to 40 years (*M* = 24.72, *SD* = 4.15). The average self-reported body mass indices (BMI = kg/m^2^) was 24.12 (*SD* = 4.39). Applying National Institute of Health [10] guidelines for classifying BMI categories, 3 (3.2%) participants were underweight (BMI < 18.5), 58 (62.4%) were in the normal weight category (BMI = 18.5 to 24.9), 27 (29%) were overweight (BMI = 25 – 29.9), and 5 (5.4%) were obese (BMI ≥30).

### Instruments

#### Trait measures (Phase 1: Pre-daily diary)

##### Demographics

This questionnaire obtained information concerning the participants’ age, height and weight.

##### Dietary restraint

The restrained eating behavior subscale of the Dutch Eating Behaviour Questionnaire (DEBQ [11]) was used to evaluate how often participants employed different dietary restraint behaviours (e.g., “Do you try to eat less at meal times than you would like to eat?”). Items from the DEBQ were rated on a 5-point Likert scale, ranging from 1 (*never)* to 5 *(very often)*, and averaged to form a single index of dietary restraint*.* The DEBQ has been shown to have good internal consistency, concurrent validity, and factorial validity [11-13]. Cronbach’s alpha for the current sample was .92. The questionnaire was included in order to evaluate the level of eating pathology in order to provide context for the results.

#### State-based measures (Phase 2: Daily diary)

##### Negative mood

The two negative mood items from the Trait Affect Scale (TAS (Blore, J., Subjective wellbeing: an assessment of competing theories, unpublished thesis)) [14] were used to measure negative state affect. The items were modified so that participants were required to indicate how they felt “right now” instead of “in general”. Items were rated on an 11-point Likert scale ranging from 0 (*not at all)* to 10 (*extremely*). Item scores were averaged so that the possible score range was 0 to 10, with higher scores reflecting greater negative mood*.* Previous research has shown this measure to be sensitive to moment-by-moment fluctuations in mood [Blore, J., Subjective wellbeing: an assessment of competing theories, unpublished thesis], and to correlate with other state-based constructs such as body dissatisfaction (e.g., (Blore, J., Subjective wellbeing: an assessment of competing theories, unpublished thesis)) [14]. Using Geldhof, Preacher, and Zyphur’s [15] method for calculating internal consistency of state-based scales in a multi-level framework, the maximal reliability for the negative mood states scale in the present study was estimated as .92.

##### Binge eating

Participants were asked whether they had consumed food since their previous assessment. An answer of ‘Yes’ was followed up with six items derived from the Questionnaire for Eating and Weight Patterns – Revised (QEWP-R [16,17]) to determine whether they had engaged in a binge eating episode. Participants received the following items reflecting DSM symptoms of a binge episode (preceded by the stem ‘*Did you experience…*’): (a) rapid consumption of food, (b) eating until uncomfortably full, (c) eating despite not being hungry, (d) eating large quantities of food (relative to your usual meal size), (e) eating alone, or (f) feeling guilt and shame after eating. Responses for these items were assigned a value of 1 for ‘Yes’ and 0 for ‘No’, so that possible scores for binge eating ranged from 0 to 6. An eating episode was classified as a probable case of binge eating if the participant reported three or more of the six symptoms listed above [1].

### Procedure

Interested participants registered for the study by following a web link (presented in recruitment presentations) that directed them to a Plain Language Statement (PLS). Those who agreed to participate were directed to a subsequent webpage to complete the baseline questionnaire. At the completion of this web-based survey, participants provided mobile phone contact details to enable the researchers to text them notifications seven times per day for a period of one week. This was automated using Red Oxygen, a bulk SMS website.

To reduce the threat of response sets due to a predictable notification schedule [18], notifications were scheduled at random intervals each day between the hours of 10 am and 8 pm, with a minimum of 1 hour between notifications. Each notification contained a web link prompting participants to complete the state-based measures. The web-survey was designed to rescale to an appropriate size for smartphones so that participants could complete the survey via either their phone or computer. Hour and date of survey completion were recorded for all samplings. At the end of the 7-day testing period, participants were mailed a $20 gift voucher as an honorarium.

### Data analytic strategy

#### Compliance to experience sampling protocol

Given the large number of assessment points used in a typical experience sampling study, it is common for participants to fail to complete some of their allotted assessments. Although missing data may lower study power, generalizability of results remain valid provided missingness is not meaningfully linked to individual difference factors [7]. Consequently, number of assessments completed (out of a total of 49) was correlated against BMI and dietary restraint, two variables that are highly relevant to the study of binge eating [19,20].

#### Threshold modeling

Hypothesis 1 was tested using logistic regression with standard errors adjusted for clustering by individual. Occurrence of a probable binge episode (coded as ‘Yes’ for binge symptoms scores greater than or equal to 3, and ‘No’ for scores less than 3) was regressed onto negative mood states, first as a continuous variable (the traditional approach), and then as a categorical variable reflecting whether a negative mood score met/exceeded a prescribed threshold value (i.e., the threshold + dose response approach). As the goal was to demonstrate the value of the threshold approach rather than derive a singular threshold value, we trialed all values of negative mood as thresholds, excluding the minimum (0) and maximum (10) values. In all models, binge eating was regressed onto the negative mood variable from the preceding assessment point in order to establish temporal precedence of the predictor variable. Moreover, time interval between assessment points was included as a covariate to control for differences in time intervals produced by the random assessment schedule. The superiority of the threshold + dose response model or the traditional model was based on which version of the predictor variable had a stronger effect size for predicting binge eating. Effect size was reported in odds ratios given that the outcome measure was dichotomous.

#### Time to onset modeling

Survival analysis was used to evaluate the extent to which level of negative mood predicted time to onset of a binge episode. Initially, a Cox proportional hazard model [21] was fitted, with negative mood as a continuous predictor; a variable to represent censoring due to completion of testing period before the onset of a binge, and participant ID as a grouping variable to allow for multiple outcomes of the same type (i.e., a binge) within the same person while correcting for non-independence that this introduces [22]. After establishing the strength and direction of linear association between severity of negative mood and time to onset, a subsequent model was run, with the same configurations as the first, with the exception that the negative mood variable was dummy coded so that more severe negative mood was compared against a less severe reference group. These dummy coded variables were introduced into the model as predictors of time to onset instead of the continuous mood variable in order to illustrate the extent to which increasing negative mood beyond a threshold level hastened the onset of a binge.

## Results

### Data cleaning and descriptives

Data were screened for patterns of missingness, presence of outliers and non-normality, and evidence of systematic bias in compliance with the experience sampling component of the study. Less than 2% missing data was found for Phase 1 (trait based measures), and dealt with using expectation maximization. On average participants completed over 80% of the experience sampling assessment points (*M* = 39.94 assessments, *SD* = 7.39, range 19–49). Individual differences in the number of Phase 2 random assessments completed were not associated reliably with BMI (*r* = −.05, *p* = .635) or dietary restraint (*r* = −.07, *p* = .497). There were no outliers or evidence of non-normality in any variable. Moreover, levels of dietary restraint (*M* = 28.78, *SD* = 8.99, possible range = 10–50) were moderate for the sample as a whole and showed considerable variability around this mean, ensuring coverage of the spectrum of severity for eating disturbances.

As may be expected given that ESM samples randomly many times per day, the prevalence of episodes of binge eating was relatively infrequent compared to the total number of assessments, occurring in 149 of 3714 assessments (4% overall across all participants). Thirty-one out of 93 participants (33%) reported at least one binge episode and, of those who reported a binge episode, the average number of binge episodes was 3.06 (*SD* = 2.46, range = 1 to 9 binge episodes). The proportion of cases that met the cut-off for the various thresholds for negative mood declined linearly, such that 94% of assessment points included a negative mood score of 1 or more, whereas only 3% of assessments included negative mood scores of 10 (see the left-hand column in Table 1).

**Table 1:** **Effect sizes (Odds Ratios) for the relationship between negative mood and binge eating**

**Predictor**	**% above**	**Odds ratio**	***p*** **value**
Traditional	n/a	1.25	.001
Threshold 1	93.8%	2.91	.038
Threshold 2	82.9%	2.37	.023
Threshold 3	56.1%	1.68	.055
Threshold 4	40.1%	1.93	.022
Threshold 5	27.8%	2.38	.003
Threshold 6	15.5%	3.68	.000
Threshold 7	8.5%	3.97	.000
Threshold 8	4.7%	4.46	.002
Threshold 9	2.8%	5.58	.002

Notes: % above = proportion of assessment points (across all participants) that met or exceeded the threshold cut-value. n/a = not applicable.

### Threshold modelling

As shown in Table 1, the association between negative mood and binge outcome was statistically significant, although the effect size was negligible when using the traditional approach of modeling negative mood as a continuous variable (odds ratio, OR = 1.25, *p* = .001). In contrast, the threshold approach more accurately predicted onset of a binge, with a linear trend in effect sizes from threshold 3 (comparing negative mood scores of 0–2 against 3–10) onwards. From threshold 6 (comparing negative mood scores of 0–5 against 6–10) onwards, the threshold approach had an effect that was at least three times as strong in predicting onset of a binge episode than the traditional approach.

### Time to onset modeling

Survival analysis established a negative log-linear association between level of negative mood and time to onset of a binge episode (*B* = −.93, *p* = .035), indicating that extreme negative mood states were temporally closer than low negative mood states to binge onset. In subsequent modeling, negative mood was dummy coded with scores of 5 (representing the midpoint on the negative mood scale) used as a reference category and values of 6 to 10 used as target group. Results of this additional analysis showed that the extent of negative mood generally heightened the onset time for a binge, with the exception of negative mood values of 9; negative mood 6 vs. 5: *B* = −.31, *p* = .020, negative mood 7 vs. 5: *B* = −.25, *p* = .039, negative mood 8 vs. 5: *B* = −.31, *p* = .086, negative mood 9 vs. 5: *B* = .13, *p* = .273, negative mood 10 vs. 5: *B* = −.16, *p* = .346.

## Discussion

### Overview of findings

Although a growing body of literature has documented the influence of negative mood states on binge eating [5], there has been limited discussion of how different modeling approaches serve to optimize prediction of binge eating. Therefore, the present study utilized the experience sampling method [7] to test alternate models of the nature of the negative mood-binge eating relationship. In particular, we tested whether the negative mood-binge eating relationship is best characterized as (i) linear or (ii) a threshold. If the latter is correct, surpassing this threshold will serve to increase both (i) risk of binge eating and (ii) subsequent negative mood. Given this, we also tested whether increased negative mood beyond the threshold predicts time to onset of the binge episode; that is, that severity of negative mood can be used to estimate time to onset of a binge.

Our findings offer support for both of our predictions. Whether or not an individual exceeded a threshold value proved to be a better predictor than the severity of negative mood for likelihood of a subsequent binge episode for all thresholds. Once the cut-point reached 6 out of 10 on the negative mood scale, the threshold version of negative mood was approximately three times stronger in predicting binge occurrence than the traditional approach of modeling negative mood severity (i.e., using mood as a continuous predictor). Model effect sizes were further optimized as the cut-point was increased.

The fact that the odds ratios continued to increase monotonically with the rising threshold cut-off rather than stabilizing or reducing may be explained in several ways. First, it may be that exceeding a threshold makes the binge outcome likely, but that continued and intensifying negative mood enhances the likelihood of a binge in much the same way that threshold dose–response models in toxicology predict that whereas sub-threshold doses of a carcinogen may have negligible and possibly contrary effects on health, once exposure exceeds the threshold, health risk is proportional to level of exposure [23,24]. Consistent with the affect regulation model [5,6], an individual when faced with negative emotional states may seek healthy solutions initially but, discouraged by continued or exacerbated symptoms, may then escalate to more unhealthy, distracting solutions, such as binge eating.

A second possible explanation for the increasing odds of a binge as the cut-point increases has to do with the timing between exposure to negative mood and onset of binge. In the survival analysis, time to onset hastened as severity of negative mood increased. Thus, whereas a negative mood state of 8 out of 10 may indicate an imminent binge episode, a moderate negative mood state (such as 6 out of 10) may allow sufficient lead time for intervening events to distract the individual, thus preventing a binge episode. Indeed, the odds of a binge episode at lower levels of negative mood may be attenuated due to naturally occurring interventions, such as presence of others, work or family responsibilities, that may take precedence and/or make it difficult or undesirable to binge. This latter (timing-based) explanation is consistent with prior studies that have shown that binge eating is preceded by a period of escalating negative mood. Crosby et al. [25] found that binge episodes were most common on days in which individuals experienced either stable elevated negative mood or consistently rising negative mood throughout the day. Similarly, Smyth et al. [26] demonstrated a steady rise in negative mood in a 6 hour period that peaked immediately prior to a binge episode.

The present findings show statistical superiority of the threshold approach for modeling likelihood of a binge. This approach may also have an advantage in interpretability over the severity approach. In the severity approach used in the present study, negative mood states were modeled as group-mean centred predictors, meaning that the substantive research question is whether deviations in self-reported mood state above one’s own usual mood state increase the likelihood of a binge eating episode. The interpretive adequacy of this approach then depends on: (1) the average mood state of the individual over the testing period; (2) the extent to which they fluctuate around this mean; and (3) how far they deviate from their mean. In the present study, 52% of participants had an average negative mood score of 3 or below. Such a low average score would mean that many instances in which the individual exceeds their average negative mood would still be comfortable levels of negative mood that are unlikely to distress them or prompt strategies such as overeating. In contrast, the threshold value can be seen to be more meaningfully interpreted as it is the level of negative mood beyond which a binge episode becomes increasingly likely.

### Implications of findings for future research and clinical practice

These findings have potential implications for treatment of binge eating. While, on the one hand, extreme negative mood states (when modeled as a threshold) are the best predictor of likelihood of a binge, it may be prudent to intervene once a patient exceeds a moderate level of negative mood for several related reasons. First, even at a threshold of 6, the mood-binge eating relationship is strong [27]. Second, as there appears to be a greater temporal gap between experience of moderate negative mood and a binge than there is between severe negative mood and bingeing, there is more time to intervene. Also, it is possible that reducing moderate negative mood to a sub-threshold level is easier than attempting to reduce severe negative mood. Third, with the advent of self-guided e-health technologies, such as interactive websites and smartphone apps that monitor risk and provide early warning signals for target event [28], the increase in false positives produced by using a less accurate threshold value may be justified if treatment is not dependent upon consultation with a clinician. As an app is more cost-effective than a therapy session and does not burden the health system with unnecessary appointments [28], a conservative approach (erring on the side of action instead of inaction) may have benefits that outweigh costs. Furthermore, the alternative – setting an extreme threshold – may increase specificity at the expense of sensitivity, as it may be that some episodes of binge eating arise when the individual’s negative mood state is only moderate.

Given that negative mood states are a common feature of many psychological disorders, including depression and bipolar disorder, this threshold model may have broader applicability for predicting symptoms of psychological disorder other than binge eating. Future research should evaluate the generalizability of our threshold approach for predicting onset of other events of clinical importance.

### Limitations

Our findings should be considered within the context of several limitations of the study. First, as effect sizes were used to compare models, it is possible that the difference in model performance (linear vs threshold) is somewhat overstated in the present study. After all, odds ratios in logistic regression analyses will be larger for categorical (threshold model) than continuous predictors (linear model). It is encouraging then, that even with this potential bias, the strength of association increased substantially as the threshold negative mood level was elevated.

Second, the method used in the present study to assess binge episodes does not definitively diagnose a binge episode. In particular, two key aspects of binge eating (quantity consumed and perceived loss of control) may not have been captured optimally. Quantity of food was assessed by self-report rather than objectively, and the five symptoms of binge eating used in the present study correspond well with perceived loss of control during eating [29], but do not explicitly ask about loss of control. Thus, it is possible that the present measure detects related, yet distinct eating behaviors, such as dysregulated or emotional eating, instead of binge eating.

A related concern is that although the sample appeared to cover the spectrum of severity of dietary restraint, formal diagnoses of eating disorders were not conducted, and given that a convenience sample of university students was recruited, it is likely that many of the participants would not meet criteria for an eating disorder diagnosis. Similarly, although we exclusively recruited women in order to be consistent with previous literature [5], binge eating is also common among men [30]. As such, it is possible that the frequency of binge eating episodes and the strength of association between negative mood and binge eating onset is weaker in the present study than would be anticipated for a clinical population that sampled both men and women. It is encouraging then, that this association yielded strong effects even in this non-clinical sample. Nevertheless, future research is needed to validate present models in a clinical sample, and this should include objective measures of binge eating occurrence.

## Conclusion

The present findings clearly support a threshold approach for modeling the influence of negative mood on likelihood of a binge episode. Even at low threshold values, this approach clearly outperformed the traditional, severity approach for predicting negative mood studies. However, the findings suggest that severity of negative mood may be more usefully employed to predict time to onset of a binge episode. Although likelihood of a binge and time to onset were strongest and shortest, respectively, at highest levels of negative mood, we argue that there is need to intervene at even moderate levels of negative mood because moderate levels were also strongly predictive of a binge outcome and, importantly, this allows more time to prevent the binge episode. This information could be used in the development of a smartphone app that seeks to disrupt the connection between negative mood and binge eating. A useful next step for future research would be to evaluate the efficacy of intervening at different threshold values for reducing the frequency of binge episodes.
